# Chemical Analysis Combined with Multivariate Statistical Methods to Determine the Geographical Origin of Milk from Four Regions in China

**DOI:** 10.3390/foods10051119

**Published:** 2021-05-18

**Authors:** Ruting Zhao, Meicheng Su, Yan Zhao, Gang Chen, Ailiang Chen, Shuming Yang

**Affiliations:** 1 Institute of Quality Standard and Testing Technology for Agro-Products, Chinese Academy of Agricultural Sciences, Beijing 100081, China; zhaoruting1201@163.com (R.Z.); sumeicheng1995@outlook.com (M.S.); chengang01@caas.cn (G.C.); ailiang.chen@gmail.com (A.C.); yangshuming@caas.cn (S.Y.); 2 Key Laboratory of Agro-Product Quality and Safety, Ministry of Agriculture, Beijing 100081, China

**Keywords:** milk, fatty acids, isotopes, mineral elements, geographical origin, multivariate statistics

## Abstract

Traceability of milk origin in China is conducive to the implementation of the protection of regional products. In order to distinguish milk from different geographical distances in China, we traced the milk of eight farms in four neighboring provinces of China (Inner Mongolia autonomous region, Hebei, Ningxia Hui autonomous and Shaanxi), and multivariate data analysis was applied to the data including elemental analysis, stable isotope analysis and fatty acid analysis. In addition, orthogonal partial least squares discriminant analysis (OPLS-DA) is used to determine the optimal classification model, and it is explored whether the combination of different technologies is better than a single technical analysis. It was confirmed that in the inter-provincial samples, the combination of the two techniques was better than the analysis using a single technique (fatty acids: R^2^ = 0.716, Q^2^ = 0.614; fatty acid-binding isotopes: R^2^ = 0.760, Q^2^ = 0.635). At the same time, milk produced by farms with different distances of less than 11 km in each province was discriminated, and the discriminant distance was successfully reduced to 0.7 km (Ningxia Hui Autonomous Region: the distance between the two farms was 0.7 km, R^2^ = 0.771, Q^2^ = 0.631). For short-distance samples, the combination multiple technologies are not completely superior to a single technique, and sometimes, it is easy to cause model over-fitting.

## 1. Introduction

With the great improvement of people’s living standard, China’s dairy farming industry has also greatly developed, and has now become the third largest producer in the world. Milk has high nutritional value, and its quality is considered to be related to the geographical location of pasture, forage, water source and other factors. Therefore, consumers pay increasingly more attention to the origin of milk, resulting in the economic value of origin information. Traceability of milk origin in China is conducive to the implementation of the protection of regional products. It can also effectively prevent the spread of food safety incidents and recall products. Therefore, the traceability of China is of high importance. Chemical fingerprinting techniques occupy an important position among all traceability methods due to its advantages of simple operation, accurate results and so on. Increasingly, the traceability of milk utilizes fatty acids, stable isotopes and mineral elements to identify the geographical origins of dairy products.

At present, many studies on the geographical origin of milk have been carried out by isotope, mineral element and fatty acid techniques. Stable isotopes are commonly used to characterize geographical origin information and to describe agricultural products’ origin information [1], where δ^2^H and δ^18^O can be used to distinguish altitude, δ^15^N can be used to determine the type of grazing vegetation and δ^13^C can determine the type of animal feed. Thus, the stable isotope ratios can be used to distinguish milk [2,3,4] and dairy products [5,6,7] of different areas.

Mineral elements have been widely used in the traceability of animal-derived agricultural products such as beef [8], pork [9], lamb [10], poultry meat [11] and honey [12,13]. This technique is also increasingly being used to identify the types and origins of milk and dairy products. In 2008, Benincasa et al. used 16 mineral elements in milk and buffalo milk to distinguish two types of milk from the same pasture [14]. In 2015, Osorio et al. determined the mineral elements in goat milk, Halloumi cheese and grazing plants in three parts of Cyprus, which can be completely distinguished, and found some mineral elements (Mn and Sr) with good traceability [15].

There have been a few studies on whether fatty acids can be used as potential chemical parameters to identify milk and dairy products of different origins. It has been reported that the proportion of fatty acids in milk produced in pastures at different latitudes varied significantly. Among them, essential fatty acids (EFA) contents and the ratios of Conjugated linoleic acid (CLA) and Polyunsaturated fatty acid (PUFA) in milk produced in mountain areas were higher than those produced in indoor cows [16]. Similar conclusions have been drawn in the study of fatty acids in milk from lowlands, mountains and highlands in Switzerland [17]. Moreover, a study of nutrients in milk from four provinces in China reported that the fatty acid contents were influenced by the geographical location [18].

Further studies showed that a model combining isotopes with mineral elements had a good differentiation effect in the geographical origin of milk, and the differentiation rate was above 90% [19,20,21]. This advantage has also been confirmed in the identification of the origins of milk, dairy products and other foods, especially in the identification of the origins of PDO foods [22,23,24,25]. In addition, in recent years, there have been studies using other technologies, such as nuclear magnetic resonance, metabolomics, infrared spectroscopy and elemental analysis, to analyze food origin. These studies have also shown that when multiple analytical techniques are combined, the results are better than when using only a single technical analysis [26,27,28,29,30,31,32].

To our knowledge, most of the research on the identification of milk producing areas has been carried out in countries or regions with far-reaching distances, such as Australia and New Zealand [1], the United States, Germany, China and France [3,4,33], or northern and central Italy [23] and northern, northwestern and southwestern China [24]. Only a few studies have focused on near-field production; in previous studies in our laboratory, Xie et al. paid attention to the traceability of milk in small-scale districts of Inner Mongolia Autonomous Region in China. It was found that a model combining all three techniques could distinguish milk samples from 11 regions in the same province and improve the accuracy of classification of a small-scale region tracking model [34]. However, in the study, Xie et al. did not verify the PCA and OPLS-DA models, which may lead to over-fitting. Although the combination of three techniques improves model accuracy, model reliability is unknown and has an impact on subsequent traceability applications. Therefore, in this study, we use permutation test to verify the model to ensure the reliability of the model.

In order to distinguish milk from different geographical distances in China, we traced the milk of eight farms in four neighboring provinces of China (Inner Mongolia autonomous region, Hebei, Ningxia Hui autonomous and Shaanxi), of which two farms in four provinces were not more than 11 km apart. We used isotopes, mineral elements and fatty acids to characterize milk origin information. Moreover, we used principal component analysis (PCA) for preliminary clustering, and further used OPLS-DA to classify milk from four provinces and distinguish milk from two farms in the province.

## 2. Materials and Methods

### 2.1. Materials

Milk samples (*n* = 120) were collected from eight large commercial farms in four provinces of China (Table 1). Milk samples were divided into three parts. One was processed according to the methods reported and used for the determination of fatty acids [18]. Two were freeze-dried for 24 h and then pulverized. The sample was mixed with a chloroform/methanol (2:1, *v*/*v*) solution at 1:5, vortexed for 10 min and centrifuged at 5000 rpm for 5 min, and the supernatant was discarded [35]. Then the previous degreasing step was repeated twice, the supernatant was discarded, the solid was retained and lyophilized to obtain a defatted dry matter (DDM) for the determination of stable isotopes and mineral elements. These samples were stored at −20 ℃ for subsequent analysis.

### 2.2. Analytical Methods

#### 2.2.1. Analysis of Fatty Acids

The samples were analyzed by an Agilent 7890A gas chromatograph with a flame ionization detector. The column is an SP-2560 (100 m × 0.25 mm × 0.20 μm; Supelco Inc., Santa Clara, CA, USA). The initial temperature is 100 °C, and raised by 5 °C min^−1^ to 210 °C, which was maintained for 25 min, then raised to 230 °C, which was held for two minutes. The injector and detector temperature were maintained at 260 °C. In total, 32 fatty acids were measured (C4:0; C6:0; C8:0; C10:0; C11:0; C12:0; C13:0; C14:0; C14:1 *cis*-9; C15:0; C15:1 *cis*-10; C16:0; C16:1 *cis*-9; C17:0; C17:1 *cis*-10; C18:0; C18:1 *trans*-9; C18:1 *cis*-9; C18:2 *cis*-6; C18:3 *cis*-6,9,12; C18:3 *cis*-9,12,15; C20:0; C20:1-*trans*-11; C20:2-*cis*11,14; C20:3-*cis*8,11,14; C20:3-*cis*11,14,17; C20:4-*cis*5,8,11,14; C22:0; C22:1-*cis*13; C22:2-*cis*13,16; C24:1-*cis*15 and CLA).

#### 2.2.2. Analysis of Stable Isotopes

For the stable isotope analysis of δ^13^C and δ^15^N, DDM and other international reference materials (USGS43, USGS40 and Sorghum Flour) were weighed into tin capsules (5 × 8 mm), and then introduced into an elemental analyzer (Flash 2000, Thermo, Waltham, MA, USA), converting the entire material into carbon dioxide and nitrogen gas analyzed by an isotope ratio mass spectrometer (Delta V Advantage of Thermo, Waltham, MA, USA). Two-point normalization of international standard materials was used. For the values of δ^13^C, USGS40 and Sorghum Flour were used for two-point normalization, and USGS43 was used for QC. For the values of δ^15^N, USGS43 and USGS40 were used for two-point normalization, and Sorghum Flour was used for QC. Blanks consisting of an empty tin capsule were included and corrections were applied to the results.

For the stable isotope ratio analysis of δ^2^H and δ^18^O, DDM and international reference materials (Caribou Hoof, Kudu Horn and EMA P2) were weighed into silver capsules (4 × 6 mm) along with other international reference materials and introduced into elemental analyzers (Flash 2000, Thermo, Waltham, MA, USA). The reactor packing is a glassy carbon reactor and silver wool. The element hydrogen and oxygen in samples were converted into H_2_ and CO at 1380 °C via pyrolysis with glass carbon. The gas was transferred to an isotope ratio mass spectrometer (Delta V Advantage, Thermo, Waltham, MA, USA). For the values of δ^2^H, Caribou Hoof and Kudu Horn were used for two-point normalization, and EMA P2 was used for QC.

#### 2.2.3. Analysis of Mineral Elements

The content of the mineral elements in DDM were determined according to published methods in our lab [36]. DDM underwent microwave digestion in a Microwave-Assisted Reaction System (MARS) (CEM, Matthews, NC, USA). A total of 0.20 g of each sample was accurately weighed directly into the PTFE digestion tube (15 mL) in triplicate, followed by the addition of 10 mL 65% HNO_3_ (analytical grade) and 1.0 mL 30% H_2_O_2_ (analytical grade) and digested for 40 min. After the sample digestion was complete, the objects in the PTFE digestion tube were transferred to a 50 mL volumetric flask, diluted with ultra-pure water, and the volume was constant to 50 mL Next, 12 elements (sodium (Na), magnesium (Mg), potassium (K), calcium (Ca), titanium (Ti), cadmium (Cr), manganese (Mn), iron (Fe), nickel (Ni), zinc (Zn), strontium (Sr) and molybdenum (Mo)) were determined by inductively coupled plasma mass spectrometry (X Series 2, Thermo Fisher, Waltham, MA, USA). Three analyses were performed for each sample and external standard analysis was performed for quantification. All results are expressed as the average of three measurements.

### 2.3. Data Processing

Multivariate statistical analysis (PCA, OPLS-DA and Permutation test) was performed on all data using SIMCA 14.1.0 software (Umetrics, Umea, Sweden). The raw data were scaled using unit variance (UV-scale), and analyzed using supervised OPLS-DA, which was used to obtain the classifying model and synchronously extract the variables with important contributions to the classification. Permutation tests were used to assess the reliability of the model.

## 3. Results and Discuss

### 3.1. Multivariate Statistical Analysis

#### 3.1.1. Identification of Milk Produced in Four Provinces

##### PCA Results

PCA is used to reduce the dimension of high dimensional variable space under the principle of minimum data information loss. These comprehensive indexes are called main components. The principal component will retain as much information as possible about the variation of the original index. In the preliminary study, single or multiple chemical parameter data (fatty acids, stable isotopes and mineral elements) were analyzed by PCA to study any possible milk clustering based on origin. PCA results (Supplementary Materials Figure S1A,B) showed that there was no obvious grouping in the score plots for inter-provincial samples, whether a single chemical parameter or the analysis with a combination of chemical parameters; however, other PCA models had no obvious classification. Thus, we consider conducting a supervised OPLS-DA of the data to improve the classification of samples.

##### OPLS-DA Results

A slight sign of classification was observed on the PCA score plot. Next, a supervised discriminant analysis of milk samples between four provinces was carried out using OPLS-DA. Moreover, we used the measure of fit of the model (R^2^) and the measure of predictive ability of the model (Q^2^) to evaluate the models.

**Figure 1:**
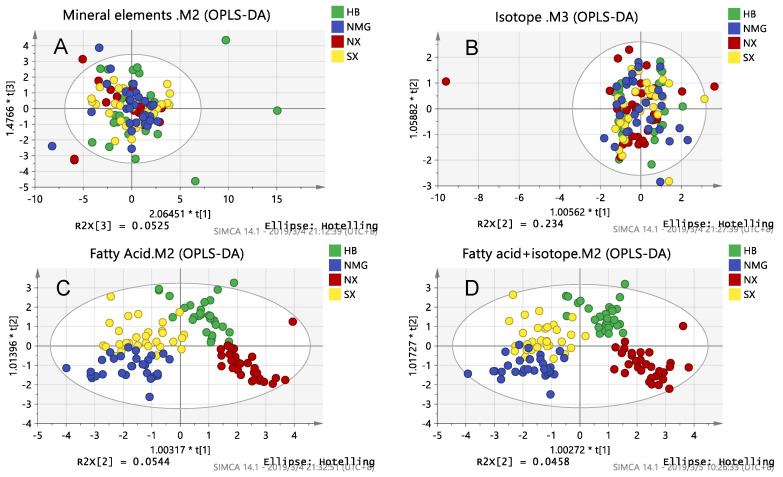
OPLS-DA score plots of inter-provincial samples obtained by a chemical analysis of: (**A**) Mineral elements; (**B**) Isotopes; (**C**) Fatty acids; (**D**) Fatty acids combined with isotopes.

There are three OPLS-DA score plots of mineral elements, isotopes, fatty acids and a combination of the best and no over-fitting in Figure 1. Four groups of milk data were analyzed by OPLS-DA. It was found that the results of the isotope and mineral element chemical parameter analysis showed no signs of classification in the score plot (Figure 1A,B). However, to our surprise, the fatty acid chemical parameter analysis showed good classification on the score plot (Figure 1C). As Figure 1C shows, Ningxia and Inner Mongolia were the most distinguished, followed by Hebei and Inner Mongolia and finally Ningxia and Shaanxi. This was because the fatty acid content and composition are affected by dairy cow breeds, feed and environmental factors such as altitude. Larsen et al. investigated the influence of regional climatic conditions on milk composition, especially fatty acid composition, and the result shows that the content of short-chain fatty acid (C4-C14), C18:0 and C18:3 n-3 are higher in central Sweden than in southern Sweden and that this is most likely because maize growing is limited to southern Sweden [37]. Thus, environmental factors affect the fatty acid content and composition in milk by affecting local plant types. Staple feed species differences (Table 1) may be the main cause of milk differences in four provinces, even more important than geographical factors. Moreover, some studies have shown that lactation also affects the fatty acid composition of milk [18,38]. Among the single techniques, the fatty acid model had the best predictive ability (Figure 1A–C). To sum up, each region in this study had a characteristic fatty acid content fingerprint and that the fatty acid chemical parameter analysis was more effective than the mineral element and isotope analysis at identifying the milk samples in the four provinces.

As shown in Table 2, the R^2^ of the isotope analysis, mineral element chemical parameter analysis and the combination of the two was less than 0.03, and the fitting degree of the model was extremely low, while the corresponding Q^2^ was negative, which indicates that the prediction ability of the models is not good [39]. Except these three models (isotopes, mineral elements, isotopes + mineral elements), the fitting degree of other models was more than 71.60%, and the prediction ability of other models was more than 56.00%. It meant that these regression models are good. Among the single techniques, the fatty acid model had the well predictive ability (R^2^ = 0.716, Q^2^ = 0.614). Among the binding technologies, fatty acid technology is helpful to improve the model prediction ability, and the binding technologies including fatty acids have better model prediction ability (fatty acid and mineral element technologies: R^2^ = 0.717, Q^2^ = 0.560; fatty acid and isotope technologies: R^2^ = 0.760, Q^2^ = 0.635; three technologies: R^2^ = 0.754, Q^2^ = 0.581). This indicated that fatty acid chemical parameters play a major role in classification, while mineral element and isotope chemical parameters are less important for classification. The results show that it is the best that the fit and prediction ability of the model combines the fatty acid with isotope technologies (R^2^ = 0.760, Q^2^ = 0.635) in four provinces, even better than that of the three technologies (R^2^ = 0.754, Q^2^ = 0.581). Similar conclusions have been reported before [40]. When R^2^ and Q^2^ of each model are close to each other, we prefer to choose a combination of multiple technologies. To sum up, we chose the model combining the fatty acid and isotope chemical parameters as the best classification model for the milk samples from the four provinces. The classification between the provinces is consistent with the results of the K-fold cross validation (Supplementary Materials Table S1).

#### 3.1.2. Identification of Milk Produced by Two Farms in the Same Province

We observed that milk samples at provincial geographic distances were differentiated significantly, so we will study the differentiation of milk samples within a smaller range. We suppose that the above methods can be used to identify the milk produced by two farms in the same province at a short distance. The analytical method used was the same as that of milk samples from different provinces.

##### PCA Analysis

For samples from two farms in Hebei, the PCA model of the isotope chemical parameter was completely divided into two categories (Supplementary Materials Figure S1C). However, no matter the other single chemical parameters or the combination of multiple chemical parameters, the score plots showed some trends of separation, though it was not completely separated. For two farm samples in Inner Mongolia and Shaanxi (Supplementary Materials Figure S1D,F), all PCA models showed a separation trend, but they were not completely separated. For two farm samples in Ningxia (Supplementary Materials Figure S1E), almost all milk samples in the model overlapped, and there was no classification trend. The above classification can be explained by the geographical distance of the two farms in the province (Table 1). The farther the geographical distance in the same province, the more obvious the classification of the samples; the closer the geographical distance in the same province, the less obvious the classification of the samples.

##### OPLS-DA Analysis

**Figure 2:**
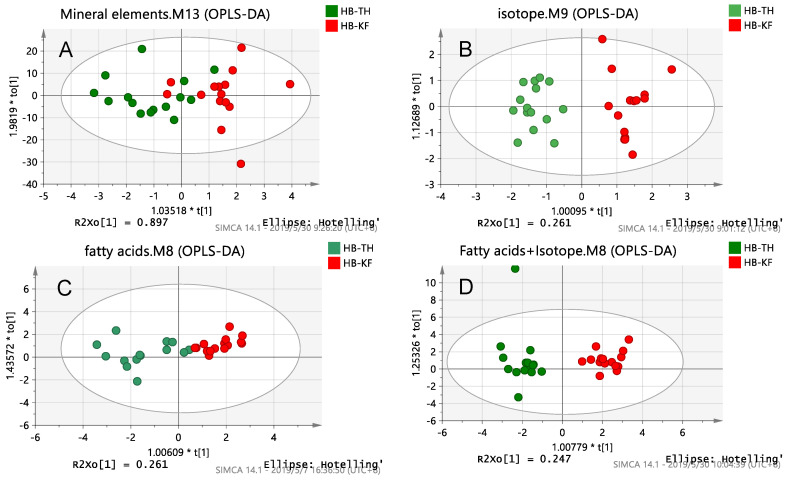
OPLS-DA score plots of Hebei samples obtained by the chemical analysis of: (**A**) Mineral elements; (**B**) Isotopes; (**C**) Fatty acids; (**D**) Fatty acids combined with isotopes.

By using OPLS-DA, a good distinction between short-distance milk in the province was obtained. There are three OPLS-DA score plots of mineral elements, isotopes, fatty acids and a combination of the best and no over-fitting in Figure 2. From OPLS-DA score plots of Hebei samples, single chemical parameters or a combination of multiple chemical parameters could be used to separate the samples from the two farms in Hebei. In the mineral element and fatty acid chemical parameter model, there were some points that were confused, which affected the classification; however, the models of the isotope chemical parameter and the combination of isotopes and other chemical parameters were well classified. Among the single techniques, the isotope model had the well predictive ability (R^2^ = 0.907, Q^2^ = 0.876). Among the binding technologies, isotope technology is helpful to improve the model prediction ability, and the binding technologies including fatty acids have better model prediction ability (mineral element and isotope technologies: R^2^ = 0.857, Q^2^ = 0.678; fatty acid and isotope technologies: R^2^ = 0.920, Q^2^ = 0.814; three technologies: R^2^ = 0.891, Q^2^ = 0.707). This indicates that isotope chemical parameters play a major role in classification, while mineral element and fatty acid parameters are less important for classification. Milk samples from two dairy farms in Hebei were fingerprinted with isotope content, which is due to the geographical specificity of the isotopes in the local plants and water. The value of δ^13^C in plants are affected by factors such as the type of plants, light, atmospheric CO_2_ concentration, temperature, air pollution and soil moisture, salinity and nutritional status, showing geographical differentiation; the value of δ^15^N in plants are influenced by parent material, soil types, topography, land use patterns and fertilization, showing geographical differentiation; the value of δ^2^H and δ^18^O of plants are related to the latitude, altitude and distance from the sea, showing geographical differentiation; the value of δ^2^H and δ^18^O of water content is affected by climate, season and precipitation, showing geographical differentiation [41,42,43]. Geographical differences in isotopes in plants and water are transferred to animals with breeding, distinguishing milk samples of different origin by determining the isotopes. As shown in Table 3, the Q^2^ of the mineral element and fatty acid chemical parameter analysis and the combination of the two were less than 0.500, which indicates that the prediction ability of the models is not good. Except these three models, the fitting degree of the other models was more than 85.70%, and the prediction ability of other models was more than 67.80%. This model combines fatty acidwith isotope chemical parameters (R^2^ = 0.920, Q^2^ = 0.814), proving to be the best classification model for milk samples in Hebei. The classification in Hebei is consistent with the results of the K-fold cross validation (Supplementary Materials Table S1).

**Figure 3:**
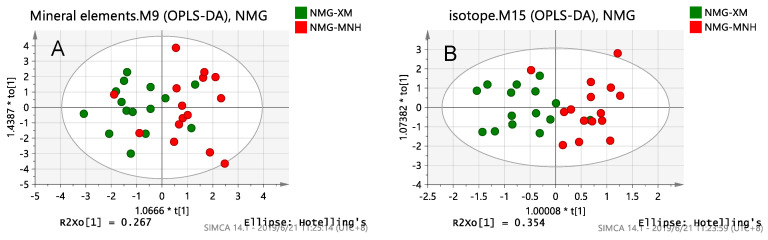

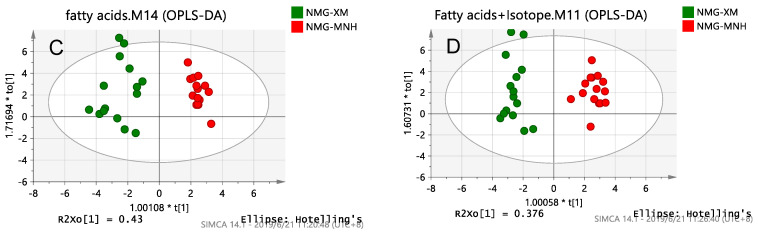
OPLS-DA score plots of Inner Mongolia samples obtained by a chemical analysis of: (**A**) Mineral elements; (**B**) Isotopes; (**C**) Fatty acids; (**D**) Fatty acids combined with isotopes.

There are three OPLS-DA score plots of mineral elements, isotopes, fatty acids and a combination of the best and no over-fitting in Figure 3. For two farms samples in Inner Mongolia, among the single-index models, the fatty acid model was the best at separating the samples. Compared with the models in Hebei, the mineral element model in Inner Mongolia confused more points, and there were a few points in the isotope model that were not distinguished. This is probably because the geographical distance between the two dairy farms narrowed from 10.7 km to 4.2 km. After combining more methods, the samples were completely separated. Among them, the model that combines all three chemical parameters further aggregated the sample points. As shown in Figure 3, each farm had a characteristic fatty acid content fingerprint and the fatty acid chemical parameter analysis was more effective than the mineral element and isotope analysis at identifying the milk samples in Inner Mongolia. For two farms samples in Inner Mongolia (Table 4), the model that combines all three chemical parameters showed the best fit and prediction ability (R^2^ = 0.985, Q^2^ = 0.910), but its y-intercepts of R^2^ was more than 0.40, which indicates that the model shows over-fitting. Thus, the model combining the fatty acid and isotope chemical parameters (R^2^ = 0.954, Q^2^ = 0.879) was determined as the best classification model for milk samples in Inner Mongolia. The classification in Inner Mongolia is consistent with the results of the K-fold cross validation (Supplementary Materials Table S1).

**Figure 4:**
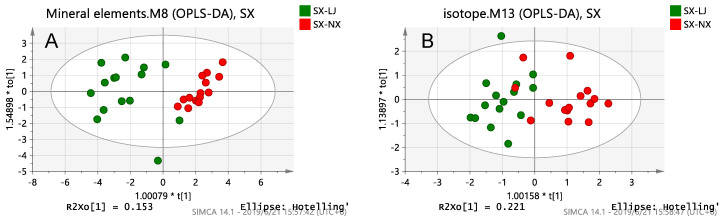

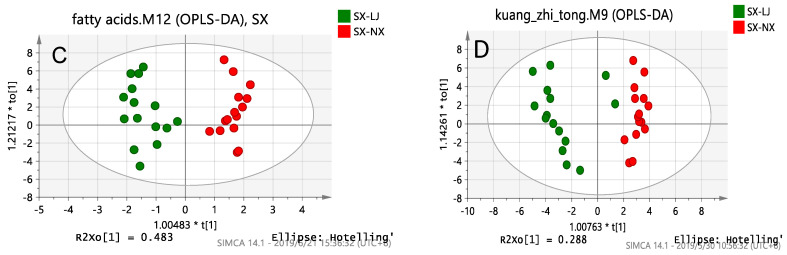
OPLS-DA score plots of the Shaanxi samples obtained by a chemical analysis of: (**A**) Mineral elements; (**B**) Isotopes; (**C**) Fatty acids; (**D**) a combination of three chemical parameters.

There are three OPLS-DA score plots of mineral elements, isotopes, fatty acids and a combination of the best and no over-fitting in Figure 4. For two farms samples in Shaanxi, among the single-index models, the fatty acid model was the best at separating the samples and the fatty acid and fatty acid-bound isotope models showed excellent separation abilities. The fitting degree of the fatty acid and fatty acids-binding isotope models of the samples in Shaanxi (Table 5) was 91.9% and 95.3%, respectively, and the prediction ability was 68.4% and 70.9%, respectively. However, the y-intercepts of R^2^ of the fatty acid-binding isotope model was more than 0.40, which indicates that the model shows over-fitting. For the other models, there were some easily confused sampling points. As shown in Figure 4, each farm had a characteristic fatty acid content fingerprint and the fatty acid chemical parameter analysis was more effective than the mineral element and isotope analysis at identifying the milk samples in Inner Mongolia. For the two farms samples from Shaanxi (Table 5), the model of fatty acid chemical parameters was the best classification model (R^2^ = 0.919, Q^2^ = 0.684). The classification in Shaanxi is consistent with the results of the K-fold cross validation (Supplementary Materials Table S1).

**Figure 5:**
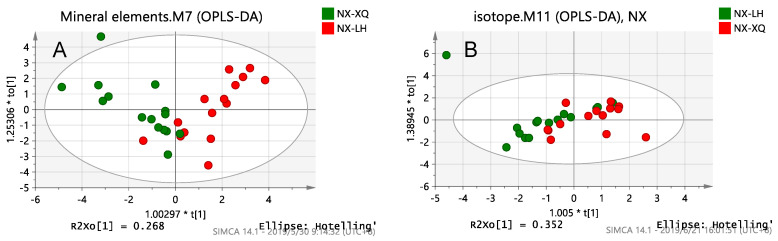

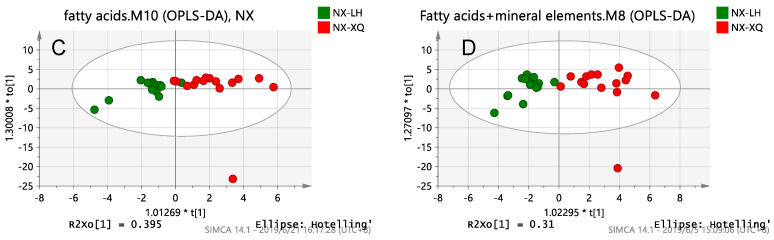
OPLS-DA score plots of the Ningxia samples obtained by a chemical analysis of: (**A**) Mineral elements; (**B**) Isotopes; (**C**) Fatty acids; (**D**) Fatty acids combined with mineral elements.

As shown in Figure 5, there are a total of four OPLS-DA scores, of which three are of mineral elements, isotopes and fatty acids parameter models, and the remaining one is a combined parameter model with the best differentiation and no over-fitting..For two farm samples in Nngxia, among the single technology models, the fatty acid and mineral element models had very good predictive ability for milksamples, but their separation abilities were far less effective than in the other three provinces (Figure 2, Figure 3 and Figure 4), because the geographical distance between the two dairy farms in Ningxia narrowed to 0.7 km. For the isotope model, there were no differentiation trends. For two farms samples in Ningxia (Table 6), only the models of fatty acid-bound element minerals (R^2^ = 0.771, Q^2^ = 0.631) showed great separation abilities. The classification in Ningxia is consistent with the results of the K-fold cross validation (Supplementary Materials Table S1).

#### 3.1.3. Validation of the OPLS-DA Model

Generally, when using this type of supervised analysis, there is a risk of over-fitting the data. Therefore, validation is crucial to verify the reliability of the model. In order to check whether the model is over-fitting, we performed the permutation test. When using the permutation test, the order of the y-variable randomly permutes the specified number 200 times, and separate models are fitted to all the permuted y-variables. Then, the original y-variable and the permuted y-variable draw a regression line. Interception is a measure of over-fitting. Desirable values of y-intercepts should be less than 0.40 for R^2^ intercept and less than 0.05 for Q^2^ intercept, respectively [44], indicating that the model is effective and there is no over-fitting. The model test results are included in each table (Table 2, Table 3, Table 4, Table 5 and Table 6).

We found that the classification models, considered good in the previous section, showed over-fitting. In the identification of inter-provincial samples, the fatty acid and fatty acid-binding isotope models (y-intercepts of R^2^ = 0.143, y-intercepts of Q^2^ = −0.348) are applicable. Thus, we still chose the model combining the fatty acid and isotope chemical parameters as the best classification model for the milk samples from four provinces. Similarly, in the two farms samples in the same province, we chose the isotope-bound fatty acid model as the discriminant model for the Hebei and Inner Mongolia samples, the fatty acid chemical parameter was selected as the discriminant model for Shaanxi and the model combining fatty acid with mineral element chemical parameters was chosen as the discriminant model for Ningxia.

## 4. Conclusions

The above research shows that multivariate statistical analysis combined with chemical parameter analysis (fatty acids, isotopes and mineral elements) can distinguish milk from different geographical distances in China. The fatty acid-binding isotope model is the best for the classification of milk samples between provinces (R^2^ = 0.760, Q^2^ = 0.635). Moreover, the model combining fatty acid with isotope chemical parameters was the best classification model for milk samples within Hebei (R^2^ = 0.920, Q^2^ = 0.814) and Inner Mongolia (R^2^ = 0.954, Q^2^ = 0.879); the models of the fatty acid chemical parameter showed great separation abilities for milk samples in Shaanxi (R^2^ = 0.919, Q^2^ = 0.684); and the model combining fatty acid with element minerals chemical parameters showed the best separation abilities for two farms samples in Ningxia (R^2^ = 0.771, Q^2^ = 0.631). In this study, traceability technology reduced the geographical distance of identified milk samples to 0.7 km. Among the five OPLS-DA models of two farms in four provinces and within the provinces, the fatty acid chemical parameter analysis was more effective than the mineral element and isotope analysis at identifying the milk samples. In addition, we found that when the sample origin distance is relatively long, the combination of the two techniques is better than the analysis using a single technique, but using the three techniques together is not superior to the combination of two technologies or a single technique, and sometimes weakens the robustness of the model. When the sample origin distance is relatively close, the combination of various technologies is not always better than a single technique, and sometimes, it can easily cause model over-fitting. These findings may be used to improve the milk traceability in China. In a future study, we will collect unknown milk samples to verify the OPLS-DA model and judge the effect of its practical application.

## Figures and Tables

**Table 1:** Information on dairy farms in four regions of China.

Origin	Number of Samples	Number of Farms	Distance between Farms (km)	North Latitude	East Longitude	Altitude (m)	Staple Feed Species
HB	30	2	10.7	38°	117°	7	Yellow corn silage, Alfalfa hay, straw
NMG	30	2	4.2	40°	111°	1030	Corn silage, Alfalfa hay, *Leymus chinensis*
SX	30	2	2.9	34°	108°	468	Corn silage, Alfalfa hay, straw
NX	30	2	0.7	37°	106°	1160	Corn silage, Alfalfa hay, cottonseed, *Leymus chinensis*

HB = Hebei Province; NMG = Inner Mongolia Autonomous Region; NX = Ningxia Hui Autonomous; Region; SX = Shaanxi Province.

**Table 2:** Characteristics and evaluations of the OPLS-DA models for the inter-provincial milk samples.

Index	FA	ISO	ME	ISO/ME	FA/ME	FA/ISO	FA/ISO/ME
R^2^	0.716	0.004	0.011	0.015	0.717	0.760	0.754
Q^2^	0.614	−0.043	−0.109	−0.088	0.560	0.635	0.581
y-intercepts of R^2^	0.112	0.031	0.061	0.045	0.179	0.143	0.211
y-intercepts of Q^2^	−0.289	−0.035	−0.614	−0.054	−0.339	−0.348	−0.417

FA = Fatty acid; ISO = Isotope; ME = Mineral elements; R^2^ = the measure of the fit of the model; Q^2^ = the measure of the predictive ability of the model.

**Table 3:** Characteristics of OPLS-DA models of milk in Hebei province.

Index	FA	ISO	ME	ISO/ME	FA/ME	FA/ISO	FA/ISO/ME
R^2^	0.755	0.907	0.562	0.857	0.768	0.920	0.891
Q^2^	0.469	0.876	−0.455	0.678	0.394	0.814	0.707
y-intercepts of R^2^	0.269	−0.020	0.170	0.281	0.374	0.315	0.396
y-intercepts of Q^2^	−0.884	−0.323	−0.182	−0.723	−0.819	−0.882	−0.826

FA = Fatty acid; ISO = Isotope; ME = Mineral elements; R^2^ = the measure of the fit of the model; Q^2^ = the measure of predictive ability of the model.

**Table 4:** Characteristics of OPLS-DA models of milk in the Inner Mongolia autonomous region.

Index	FA	ISO	ME	ISO/ME	FA/ME	FA/ISO	FA/ISO/ME
R^2^	0.919	0.599	0.410	0.763	0.955	0.954	0.985
Q^2^	0.876	0.530	−0.243	0.432	0.813	0.879	0.910
y-intercepts of R^2^	0.233	0.057	0.265	0.353	0.559	0.388	0.677
y-intercepts of Q^2^	−0.680	−0.306	−0.339	−0.718	−1.130	−1.060	−1.560

FA = Fatty acid; ISO = Isotope; ME = Mineral elements; R^2^ = the measure of the fit of the model; Q^2^ = the measure of predictive ability of the model.

**Table 5:** Characteristics of OPLS-DA models of milk in Shaanxi province.

Index	FA	ISO	ME	ISO/ME	FA/ME	FA/ISO	FA/ISO/ME
R^2^	0.919	0.673	0.773	0.725	0.810	0.953	0.839
Q^2^	0.684	0.548	0.602	0.688	0.685	0.709	0.721
y-intercepts of R^2^	0.371	0.058	0.134	0.035	0.240	0.417	0.301
y-intercepts of Q^2^	−0.873	−0.300	−0.321	−0.303	−0.557	−1.020	−0.563

FA = Fatty acid; ISO = Isotope; ME = Mineral elements; R^2^ = the measure of the fit of the model; Q^2^ = the measure of predictive ability of the model.

**Table 6:** Characteristics of OPLS-DA models of milk in the Ningxia Hui autonomous region.

Index	FA	ISO	ME	ISO/ME	FA/ME	FA/ISO	FA/ISO/ME
R^2^	0.630	0.310	0.654	0.474	0.771	0.557	0.777
Q^2^	0.434	−0.601	0.407	0.416	0.631	0.393	0.596
y-intercepts of R^2^	0.139	0.011	0.176	0.137	0.328	0.232	0.434
y-intercepts of Q^2^	−0.833	−0.217	−0.393	−0.257	−0.704	−0.453	−0.666

FA = Fatty acid; ISO = Isotope; ME = Mineral elements; R^2^ = the measure of the fit of the model; Q^2^ = the measure of predictive ability of the model.

## Data Availability

Data are contained within the article or Supplementary Materials.

## Supplementary Materials

The following are available online at https://www.mdpi.com/article/10.3390/foods10051119/s1, Figure S1: PCA score plots for each technical model: (A, B) Isotope model and isotope-binding fatty acid model for interprovincial samples; (C–F) Isotope-bound fatty acid models of provincial samples, Table S1: Accuracy of OPLS-DA models for milk discrimination between provinces and within the same provinces.
