# Supplementary material for: Chemical Analysis Combined with Multivariate Statistical Methods to Determine the Geographical Origin of Milk from Four Regions in China
Source: Foods. 2021 May 18;10(5):1119. doi: 10.3390/foods10051119 (PMC8158098; doi:10.3390/foods10051119)
Supplement: Supplementary file 1 [file foods-10-01119-s001.zip › foods-1205357-supplementary.pdf]

Table S1. Accuracy of OPLS-DA models for milk discrimination between provinces and within the same provinces

Chemical techniques	Four regions	Within the province			
		HB	NMG	SX	NX
FA	94.17%	93.33%	100%	100%	93.33%
ISO	29.17%	100%	90.33%	90%	73.33%
ME	24.17%	83.33%	83.33%	93.33%	93.33%
ME/ISO	27.50%	100%	96.67%	93.33%	90.00%
FA/ISO	96.67%	100%	100%	100%	90.33%
FA/ME	95.83%	100%	100%	93.33%	100%
FA/ME/ISO	97.50%	100%	100%	93.33%	100%

FA = Fatty acid; ISO =Isotope; ME = Mineral elements

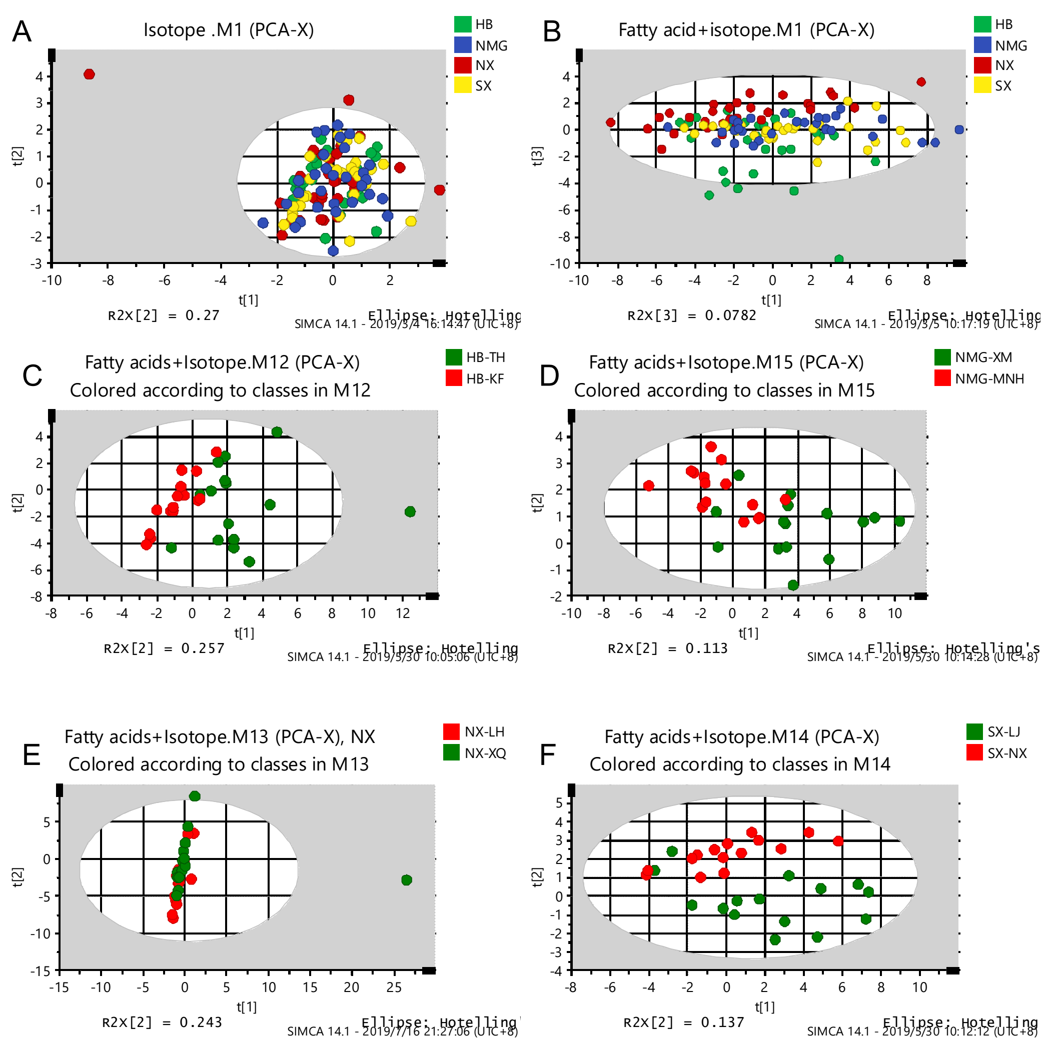

Figure S1. PCA score plots for each technical model：(A/B) Isotope model and isotope-binding fatty acid model for interprovincial samples; (C/D/E/F) Isotope-bound fatty acid models of provincial samples
